# Editorial: Highlights from the 12th plant growth-promoting rhizobacteria workshop

**DOI:** 10.3389/fpls.2024.1470576

**Published:** 2024-08-14

**Authors:** Choong-Min Ryu, Louise M. Nelson, Luz de-Bashan

**Affiliations:** ^1^ Infectious Disease Research Center, Korea Research Institute of Bioscience and Biotechnology (KRIBB), Daejeon, Republic of Korea; ^2^ Department of Biosystems and Bioengineering, Korea Research Institute of Bioscience and Biotechnology, University of Science and Technology, Daejeon, Republic of Korea; ^3^ Department of Cell and Developmental Biology, University of California, San Diego, San Diego, CA, United States; ^4^ Department of Pediatrics, School of Medicine, University of California, San Diego, San Diego, CA, United States; ^5^ Department of Biology, University of British Columbia (Okanagan), Kelowna, BC, Canada; ^6^ The Bashan Institute of Science, Auburn, AL, United States; ^7^ Department of Entomology and Plant Pathology, Auburn University, Auburn, AL, United States

**Keywords:** PGPR, induced systemic resistance, induced systemic tolerance, microbiome, LMICs, PGPB

## Introduction

The term ‘Plant Growth-Promoting Rhizobacteria (PGPR)’ was coined by Joseph W. Kloepper in 1978 (Kloepper and Schroth, 1978). He defined PGPR as “Naturally-occurring, root-colonizing bacteria that benefit plants by growth promotion and biocontrol”. Nine years later, he organized the first international PGPR workshop at 1987 in Canada. Subsequently, the Workshop has been held in Switzerland (1990), Australia (1994), Japan (1997), Argentina (2000), India (2003), The Netherlands (2006), Oregon (2009), Colombia (2012), Belgium (2015), and Canada (2018). The International PGPR Workshop series is the most comprehensive conference in the field of PGPR/Plant Growth-Promoting Bacteria (PGPB). Professor Kloepper’s philosophy for the workshops was as a venue to bring together young and established researchers and industry to increase our understanding of the fundamental and applied aspects of PGPR via formal presentations and informal discussion to advance the field. The 12th PGPR Workshop was held in Toulouse, France, on May 29th - June 2nd, 2023. This Frontiers Research Topic aims to collect selected contributions from this PGPR Workshop. This Research Topic aims to bring together new findings and applications of PGPR.

## Carrying the old knowledge gained into new fields

Since Professor Kloepper’s retirement, the chronicles of a representative PGPR strain were summarized by former students in his laboratory at Auburn University (Jang et al.). Out of many PGPR species, *Bacillus* spp. received much attention due to their ability to form endospores that are resistant to biotic and abiotic stresses such as limited nutrients, hydration, heat, and extreme pH. *Bacillus velenzensis* (formerly *Bacillus subtilis* and *B. amyloliqufaciens*) strain GB03 was the first bacilli product developed by long-term collaboration with Professor Kloepper and Gustafson LLC, Plano Texas, USA. In this review, authors describe the process from isolation to commercialization as well as the mechanisms of plant growth promotion and biological control against soil-borne pathogens. The status of PGPR study in central Africa including Burundi, Rwanda, and the Democratic Republic of Congo was reported by Nihorimbere and colleagues (Nihorimbere et al.). PGPR provide a greater potential than chemical pesticides in the tropical region to manage the plant diseases occurring because the European Union banned 30% of the pesticides used in this region. The transfer of knowledge and know-how from scientists in global north countries needs to come through scientific collaboration.

## Make plants healthier in the face of abiotic stresses

As we already recognized in 2023, the hottest year in human history reported by European Union’s Copernicus Climate Change Service (https://www.ncei.noaa.gov/access/monitoring/monthly-report/global/202313), the crisis of climate change is upon us. Due to climate change, the cultivation of crop plants can be limited. To combat environmental stresses, we need silver bullets to overcome these constraints. Elicitation of induced systemic tolerance by PGPR can be a solution to abiotic stresses in plants (Yang et al., 2009). Through biochemical and molecular evaluation, three bacterial strains isolated from tomato soil and roots were selected for plant responses under water stresses. The tomato’s physiological changes were characterized following treatment with PGPR (Zampieri et al.). Heavy metals can also suppress plant growth directly. A metal tolerant PGPR strain *Paraburkholderia ultramafica* STM0279^T^ elicited metal tolerance in a tropical hub plant *Tetraria comosa* (Bourles et al.). The experiments were conducted in New Caledonian ultramafic soil also known as serpentine soil containing high level of toxic metals, Ni, Co, Cr, and Mn and low levels of essential plant nutrients N, P, and K. *T. comosa*, belonging to the Cyperaccae family, is a pioneer and abundant herbaceous member in New Caledonian ultramafic soil. Abou Jaoude et al. gave a new insight into PGPR and their metabolite-induced changes to plant photosynthetic activity as a parameter of plant ecophysiology under non-stress and biotic and abiotic stress conditions through metanalyses of the topic-related references. Collectively, PGPR provide potential to protect plants from environmental stresses derived from climate change.

## Protecting plants against unculturable pathogens

PGPR utilize plant protectants as biological control tools against foliar and soilborne pathogens. Due to the experimental difficulty of growing non-culturable pathogens, testing the biocontrol efficacy by PGPR was limited. Here, two new results with unculturable aboveground and belowground pathogens shed light on a new strategy to control non-culturable and economically important pathogens. First, the cell wall degrading enzyme LytD from *B. subtills* plays a pivotal role in biological control against grape downy mildew caused by *Plasmopara viticola* (Wang et al.). The loss of function of the autolysin *N*-acetylglucosaminidase LytD in *B. subtills* GLB191 disrupts biocontrol capacity, biofilm formation, and leaf colonization. The authors provide the first example of the role of bacterial autolysin in plant protection by PGPR. Secondly, a group of scientist in Colombia targeted the management of another non-culturable fungal pathogen *Plasmodiopora barassicae*, a causal factor of clubroot diseases in cruciferous plants. The PGPR consortium reduced the disease incidence up to 56% indicating an efficient and integrated disease management tool even in the highly *P. barassicae*-infested fields (Moreno-Velandia et al.).

## Beyond growth promotion, improving plant metabolite and mineral contents

Tanney et al. demonstrated that application of *Bacillus* sp., *Pseudomonas* sp., and two bacterial combination treatments increased the cannabinoid contents of cannabis (*Cannabis sativa*). Cannabis has received increased attention recently, although heavy legalization limits its cultivation under greenhouse and field conditions. PGPR inoculation triggered trichome development and increased cannabinoid amount only under a low nutrient regime for six weeks (Tanney et al.). In addition to PGPR-mediated increase of plant secondary metabolites, it is also reported that mineral uptake into vegetables was promoted by *B. subtilis* inoculation (Oliveira et al.). *B. subtilis* inoculation increased Ca, Mg, S, K, and N in shoot and also enhanced photosynthesis, intracellular CO_2_ contents, and water use efficiency. Guardado-Fierros et al. compared a classic indole-3- 202 acetic acid (IAA) quantification method, the Salkowski method to 203 ultra-performance liquid chromatography coupled with a Mass 204 Spectrometer (LC-MS/MS) using the supernatant culture of ten 205 PGPR strains. The Salkowski method overestimated the IAA concentration by a maximum of 1,042 fold without tryptophan and 16,330 fold with tryptophan due to the detection of other indole compounds. The demands for high quality and functional food are increasing worldwide. PGPR bring a great potential to induce beneficial plant secondary metabolite production or mineral uptake to meet consumer demand.

## Unveiling genetic architecture of PGPB-triggered growth promotion

To date, plant genetic traits associated with PGPB-triggered plant growth promotion have not been widely assessed. The PGPB *Pseudomonas siligins* OTU6_*Psi*_1 isolated from *Arabidopsis thaliana* leaf was examined in genome wide association studies by inoculating seed of 162 natural accessions from the southwest of France under *in vitro* conditions (Ramirez-Sanchez et al.). From intensive analysis, the polygenic genetic architecture underlying a strong negative trade-off was detected. Besides sole growth-related genes, dual functionality genes, *SBT3.5* and *JAZ11* for growth and defense were identified. The results will help breeding programs improve the capacity for PGPR-induced crop growth promotion, yield and quality.

## Outlook

This Research Topic covered recent data on the PGPR/PGPB-plant interactions. PGPR/PGPB are recognized as a potential silver bullet to reduce these detrimental side effects while sustainably enhancing crop production. The current studies pave a new way for next-generation PGPR applications. We are very positive about the future of PGPR-related fields. Strong collaboration between industry and academia and between global south and north is also required now. Finally, as was Professor Kloepper’s wish, we hope to inspire the next generation of researchers to inherit this field and to share new ideas to solve the impact of planetary problems such as climate change on agriculture.
